# Adverse events during intrahospital transport of critically ill patients: incidence and risk factors

**DOI:** 10.1186/2110-5820-3-10

**Published:** 2013-04-12

**Authors:** Erika Parmentier-Decrucq, Julien Poissy, Raphaël Favory, Saad Nseir, Thierry Onimus, Mary-Jane Guerry, Alain Durocher, Daniel Mathieu

**Affiliations:** 1 Service d’Urgence Respiratoire, Réanimation Médicale et Medecine Hyperbare, Université de Lille II et Centre Hospitalier et Universitaire de Lille, Lille 59037, France

**Keywords:** Intrahospital transport, Adverse events, Mechanical ventilation, Critical care, Risk factors

## Abstract

**Background:**

Transport of critically ill patients for diagnostic or therapeutic procedures is at risk of complications. Adverse events during transport are common and may have significant consequences for the patient. The objective of the study was to collect prospectively adverse events that occurred during intrahospital transports of critically ill patients and to determine their risk factors.

**Methods:**

This prospective, observational study of intrahospital transport of consecutively admitted patients with mechanical ventilation was conducted in a 38-bed intensive care unit in a university hospital from May 2009 to March 2010.

**Results:**

Of 262 transports observed (184 patients), 120 (45.8%) were associated with adverse events. Risk factors were ventilation with positive end-expiratory pressure >6 cmH_2_O, sedation before transport, and fluid loading for intrahospital transports. Within these intrahospital transports with adverse events, 68 (26% of all intrahospital transports) were associated with an adverse event affecting the patient. Identified risk factors were: positive end-expiratory pressure >6 cmH_2_O, and treatment modification before transport. In 44 cases (16.8% of all intrahospital transports), adverse event was considered serious for the patient. In our study, adverse events did not statistically increase ventilator-associated pneumonia, time spent on mechanical ventilation, or length of stay in the intensive care unit.

**Conclusions:**

This study confirms that the intrahospital transports of critically ill patients leads to a significant number of adverse events. Although in our study adverse events have not had major consequences on the patient stay, efforts should be made to decrease their incidence.

## Background

Management of critically ill patients in the intensive care unit (ICU) requires investigations and therapeutic procedures leading to numerous transports outside the ICU. These intrahospital transports (IHT) are at risk of complication and should be considered as an important part of the ICU risk management program. However, an adverse event (AE) during transport remains common and may induce an important risk for the patient. This risk has to be evaluated by the physician before ordering a diagnostic or therapeutic procedure, based on a benefit/risk analysis in which the risk of the IHT has to be put in balance with the expected benefit of the procedure [1]. Thus, reducing the risk of IHT adverse events is essential to ICU patient management.

Many recommendations are available [2-6], stemming from personal experience and expert opinion [4,7]. Several authors have identified effective "protective” factors for limiting AE, such as regular patient [8-12] and equipment checks [4,11] during IHT, meticulous preparation of the patient, appropriate sedation [4], a specialised and experienced escort [4,11], correct use of protocols [13], and diagnostic and therapeutic units located within easy reach of the emergency department or ICU [4,11]. Also, good clinical sense is required to decrease AE during IHT [1].

Incidence and severity of AE vary according to studies [8,9,11,14]. These discrepancies may be explained at least in part by differences in definition. The most clinically useful definition of major AE is one that leads to a change of therapy during IHT [13]. Discrepancies also may be explained by the time-period studied: AE can arise during transport or secondarily. Finally, IHT have been suspected to be one of the causes of ventilator-associated pneumonia and their occurrence also should be studied [15].

The main objectives of this observational study were first to determine the frequency and risk factors for AE during IHT of critically ill patients and, second, to determine the consequences of these AE during IHT and what improvements could be put in place in our ICU.

## Methods

This prospective, observational study was conducted in a 38-bed medical ICU in a regional and teaching hospital in France from May 1, 2009, to March 21, 2010. The Ethics Committee of the Institutional Review Board for the University Hospital of Lille approved the design of our study. Because of the noninterventional nature of our study, patient consent was waived.

### Patients

Every mechanically ventilated patient who needed IHT for a computed tomography (CT) scan was enrolled. ICU admission before May 1, 2009, ICU discharge after March 21, 2010, IHT for therapeutic procedures, and IHT for investigations other than a CT scan were exclusion criteria.

### Methods

#### IHT

Before this study was undertaken, a specific protocol for managing IHT was in place in our ICU to limit AE (Additional file 1). Our protocol was in accordance with recommendations in effect at the time of the study and included regular equipment and patient checks [4]. The mechanically ventilated patients were accompanied by a transport team composed of a resident and a porter. The resident is the junior physician directly involved in the daily care of the patient. All residents receive specific training regarding IHT when they start their 6-month training period in the ICU. The four porters have been working in our ICU for many years and have significant experience with IHT of mechanically ventilated patients. During IHT, portable devices with settings adjusted to clinical necessity are used to monitor vital signs.

#### Data collection

Clinical patient characteristics and IHT characteristics were prospectively recorded. Data were collected through a case report form in part by the porter for nonmedical data and by the resident for medical data and adverse events during IHT. This case report form was created for the study and also included the transport protocol of our ICU. Case report forms were stocked with airway equipment. So for every IHT of ventilated patient, a case report form was distributed by the porter and recovered within 1 hour after patient return to his room when storing airway equipment. All case report forms were recovered by one of the investigators each day.

The following AE were prospectively recorded for all IHT: agitation, accidental pulling out of nasogastric tube, vomiting, peripheral venous catheter incident (accidental dislodgment, disconnection, or thrombosis), central venous catheter incident (disconnection or thrombosis), arterial line incident (disconnection or thrombosis), accidental dislodging of urinary catheter, disconnection of endotracheal tube, and airway equipment were considered minor patient-related AE; oxygen desaturation (pulse oximetry (SpO_2_) <95% or >5% decrease in SpO_2_ for more than 1 minute) [16], accidental extubation, accidental central venous catheter removal, disconnection of chest tube, severe hypotension (systolic blood pressure inferior than 90 mmHg or 20 mmHg decrease in systolic or diastolic blood pressure more than 1 minute) [11,17], arrhythmia, cardiac arrest were considered major patient-related AE; incidents with airway equipment (alarms, transport ventilator malfunction, or problems with oxygen supply), battery supply problems with the monitor or with infusion pumps were considered equipment-related incidents [8]. Change of therapy during IHT was noted. Fluid challenge was defined by 500 ml of crystalloid or colloid administration. After IHT, subsequent events, such as acute respiratory distress syndrome or ventilator-associated pneumonia, also were recorded. The diagnosis of ventilator-associated pneumonia was made according to the 2005 American Thoracic Society guidelines [18].

#### Data analysis

Statistical analysis was performed using SPSS software (version 15.0, SPSS, Chicago, IL). Quantitative values were expressed in median with 25th and 75th percentiles and comparisons between groups were made using Mann–Whitney *U* test. Qualitative data were expressed as values and percentages and compared by Chi-square test. A value of *p* < 0.05 was considered significant. For discrete numerical values, such as positive end-expiratory pressure (PEEP) level, the optimal threshold value was determined by ROC curve.

Risk factors for AE during IHT were tested first by univariate analysis. Those with a significance level of *p* < 0.1 were included in a logistic regression with Wald method analysis as independent variables. Results were reported as odds ratios (OR), and statistical significance was ascertained by the 95% confidence interval.

## Results

### Patients

**Figure 1:**
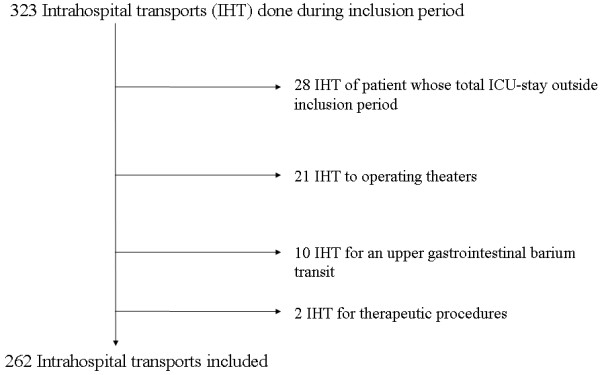
Flowchart.

During the inclusion period, 753 patients were hospitalized in our ICU, for whom 323 IHT were carried out. We recorded and analysed 262 IHT of 184 patients based on the inclusion/exclusion criteria (Figure 1). Population characteristics are shown in Table 1.

**Table 1:** Global characteristics of patients

**All patients (n = 184)**	**Median**	**[25**^**th**^**-75**^**th **^**percentiles]**	**Number**	**%**
**Characteristics of patients**				
Age (yr)	58	[48–71]		
Man			129	70.1
SAPS II	48	[34–62]		
COPD			26	14
ALI			125	68
ARDS			56	30.4
**Characteristics of hospitalization in ICU**				
Length of stay in ICU (days)	16	[8.25-25.75]		
Length of mechanical ventilation (days)	11	[6–21.75]		
Dialysis during hospitalization			48	26
Ventilator-associated pneumonia			56	30.4
Death Day 28			55	29.9
Death in ICU			69	37.5

*SAPS II* = Simplified Acute Physiology Score II; *COPD* = chronic obstructive pulmonary disease; *ALI* = acute lung injury; *ARDS* = acute respiratory distress syndrome.

A total of 128 patients underwent only one IHT during their hospitalization in ICU, 41 patients underwent two transports, 10 patients underwent three, 4 patients underwent four transports, and one patient underwent six IHT for CT scans. Seventeen IHT were done outside of working hours (between 9 p.m. and 7 a.m.). Patient data before and during IHT are shown in Table 2.

**Table 2:** Characteristics of patients before and during IHT

**All intrahospital transports (n = 262)**	**Median**	**[25**^**th**^**-75**^**th **^**percentiles]**	**Number**	**%**
**Characteristics of patients before IHT**				
**SOFA the day of IHT**	**5**	[3-8]		
**Endotracheal tube**			**262**	**100**
Orotracheal tube			169	64.5
Nasotracheal tube			64	24.4
Tracheotomy			29	11.1
**Central venous catheter**			**220**	**84**
Subclavian vein			148	56.5
Internal jugular vein			23	8.8
Femoral vein			49	18.7
**Arterial line**			**197**	**75.2**
Radial			159	60.7
Femoral			38	14.5
**Nasogastric tube**			**242**	**92.4**
**Urinary catheter**			**255**	**97.3**
**Chest tube**			**35**	**13.4**
**Inhaled nitric oxide**			**4**	**1.5**
**Ventilatory mode before IHT**				
VAC			214	81.7
BIPAP			18	6.9
PSV			27	10.7
**FiO2 before IHT (%)**	**45**	**[35–60]**		
**PEEP before IHT (cmH**_**2**_**O)**	**6**	[5-8]		
**Treatments before IHT**				
Sedation			153	58.4
Neuromuscular blocker			27	10.3
Norepinephrine			45	17.2
Dobutamine			32	12.2
Epinephrine			4	1.5
**Characteristics of patients during IHT**				
**Number of infusion pumps**	**2**	[2,3]		
**Duration of CT scan (min)**	**30**	**[20–40]**		
**Duration of transport (min)**	**50**	**[40–60]**		
**Ventilatory mode during transport**				
VAC			249	95.0
PSV			13	5.0
**Ventilatory parameters during transport**				
Ventilatory mode change for transport			35	13.4
FiO2 during IHT (%)	100	[50–100]		
PEEP during IHT (cmH_2_O)	6	[5-8]		
Change of PEEP for transport			23	8.8
**Treatment modification for transport**			**59**	**22.5**
Sedation for transport			38	14.5
Neuromuscular blocker use for transport			16	6.1
Fluid challenge for transport			12	4.6
**Junior physician accompanying IHT**				
Junior physician with experience			144	60
Junior anesthetist physician			121	50.4

*SOFA* = Sepsis-related Organ Failure Assessment; *SAPS* II = Simplified Acute Physiology Score II; *IHT* = intrahospital transport; *VAC* = volume assist–control; *BIPAP* = bi-level positive airway pressure; *PSV* = pressure support ventilation; *FiO*_2_ = fraction of inspired oxygen; *PEEP* = positive end-expiratory pressure.

### Recorded adverse events

We recorded 120 IHT with one or more AE (45.8% of transports; Table 3). Eighty-six equipment-related incidents were noted (32.8% of all IHT). Sixty-eight IHT were patient-related AE (26% of IHT), within 44 were major patient-related AE (16.8% of IHT). These were predominantly oxygen desaturation (23 cases or 8.8% of IHT) and hemodynamic instability (13 cases or 5% of IHT). No cardiac arrest occurred during IHT.

**Table 3:** Adverse events during transport

**All intrahospital transports (n = 262)**	**Number**	**%**
**Adverse events during transport**	**120**	**45.8**
**Patient-related adverse events during transport**	**68**	**26**
**Major patient-related adverse events during transport**	**44**	**16.8**
Oxygen desaturation	23	8.8
Extubation	1	0.4
Accidental central venous catheter removal	1	0.4
Hemodynamic instability	13	5
Increased vasopressor dose	5	1.9
**Minor patient-related adverse events during transport**	**53**	**20.2**
Agitation	38	14.5
Accidental nasogastric tube pull out	1	0.4
Vomiting	3	1.1
Peripheral venous catheter incident	4	1.5
Central venous catheter incident	6	2.3
Arterial line incident	5	1.9
Accidental dislodging of urinary catheter	1	0.4
Disconnection of endotracheal tube and airway equipment	2	0.8
**Equipment-related incidents during transport**	**86**	**32.8**
Incident with airway equipment (alarm, adjustment)	46	17.6
Incident with monitor (battery, alarm)	45	17.2
Incident with infusion pumps (battery, alarm)	18	6.9

### Risk assessment before transport

#### Total adverse events

We examined which parameters before transport could predict AE during IHT. All data collected were considered as potential risk factors (Table 4). The optimal threshold value determined by ROC curve was 6 for PEEP level (sensibility = 0.6, specificity = 0.76).

**Table 4:** Risk factors of adverse events during IHT

**Qualitative data**		**Univariate analysis**		**Multivariate analysis**
	**OR (95% CI)**	***p***	**OR (95% CI)**	***p***
**Treatments before transport**				
Dialysis	0.76 (0.44-1.32)	NS		
Sedation	2.02 (1.22-3.34)	0.008	1.85 (1.1-3.11)	0.021
Neuromuscular blockers use	1.55 (0.69-3.45)	NS		
Norepinephrine before transport	1.61 (0.84-3.06)	NS		
Dobutamine before transport	1.21 (0.58-2.54)	NS		
**Ventilatory mode before IHT**				
VAC	0.9 (0.48-1.69)	NS		
BIPAP	0.94 (0.36-2.47)	NS		
PSV	1.42 (0.65-3.11)	NS		
FiO2 before IHT >60%	1.51 (0.81-2.83)	NS		
PEEP before IHT >6 cmH_2_O	2.27 (1.34-3.85)	0.003	2.28 (1.32-3.95)	0.003
**Transport**				
More than four infusion pumps for transport	3.4 (0.88-13.03)	0.071		NS
Ventilatory mode change for transport	1.3 (0.64-2.65)	NS		
Change of PEEP for transport	0.84 (0.05-13.64)	NS		
Treatment modification for transport	2.22 (1.23-4.01)	0.011		NS
Sedation for transport	1.77 (0.88-3.55)	NS		
Neuromuscular blocker use for transport	1.2 (0.44-3.29)	NS		
Fluid challenge for transport	6.36 (1.37-29.64)	0.014	6.48 (1.32-31.69)	0.021
**Quantitative data**			**Univariate**	**Multivariate**
	**Without AE**	**With AE**	**analysis**	**analysis**
**Patient data (Med (25**^**th **^**,75**^**th **^**))**				
Age (yr)	61 (44–74)	58 (50.5-68)	NS	
SAPS II	48 (34–61)	48 (34–64)	NS	
**Transport's data (Med (25**^**th **^**,75**^**th **^**))**				
SOFA the day of transport	5 [3-7]	5 [3-8]	NS	
MV duration before transport (days)	4 [2–11.25]	4 [2–10.75]	NS	
No. of infusion pumps during transport	2 [1-3]	3 [2,3]	p = 0.015	NS

*VAC* = volume assist–control; *BIPAP* = bi-level positive airway pressure; *PSV* = pressure support ventilation; *FiO*_*2*_ = fraction of inspired oxygen; *PEEP* = positive end-expiratory pressure; *SOFA* = Sepsis-related Organ Failure Assessment; *SAPS II* = Simplified Acute Physiology Score; *MV* = mechanical ventilation; *OR* = odds ratio; *CI* = confidence interval.

In univariate analysis, AE during IHT were associated with: sedation before transport, PEEP > 6 cmH_2_O, high number of infusion pumps, treatment modification for transport, and particularly fluid challenge. In multivariate analysis, increased risk of AE was associated with fluid challenge for transport (OR = 6.5 [1.3-31.7]; *p* = 0.021), PEEP > 6 cmH_2_O (OR = 2.28 [1.3-4]; *p* = 0.003), sedation before transport (OR = 1.85 [1.1-3.1]; *p* = 0.021). Severity scores (SAPS II at admission or SOFA score the day of HIT) were not found to predict AE during transport. The repetition of IHT for the same patient is not a risk factor for AE during IHT. AE during IHT were not associated with the time of day or the day of the week that IHT was done.

#### Patient-related adverse events

We counted 68 patient-related AE (26% of IHT). With univariate analysis, risk factors for these events were: treatment modification for transport (OR = 2.7 [1.4-4.9]; *p* = 0.002), such as increasing sedation for IHT (OR = 3.1 [1.5-6.2]; *p* = 0.003), and PEEP > 6 cmH_2_O (OR = 2.2 [1.2-3.8]; *p* = 0.01). With multivariate analysis, only PEEP > 6 cmH_2_O (OR = 2.1 [1.2-3.8]; *p* = 0.01) and treatment modification for transport (OR = 2.8 [1.5-5.3]; *p* = 0.001) were found to be significant (Table 5).

**Table 5:** Risk factors of patient-related adverse events during IHT

**Qualitative data**	**Univariate analysis**			
	**OR (95% CI)**	***p***	**OR (95% CI)**	***p***
**Treatments before transport**				
Dialysis	0.84 (0.45-1.58)	NS		
Sedation	1.48 (0.83-2.61)	NS		
Neuromuscular blockers use	0.78 (0.3-2.02)	NS		
Norepinephrine before transport	1.71 (0.86-3.39)	NS		
Dobutamine before transport	1.11 (0.49-2.53)	NS		
**Ventilatory mode before IHT**				
VAC	1.09 (0.53-2.24)	NS		
BIPAP	1.44 (0.52-3.99)	NS		
PSV	0.74 (0.29-1.91)	NS		
FiO2 before IHT > 60%	1.52 (0.77-2.99)	NS		
PEEP before IHT > 6 cmH_2_O	2.16 (1.22-3.81)	0.01	2.11 (1.17-3.8)	0.01
**Transport**				
More than four infusion pumps for transport	1.07 (0.28-4.17)	NS		
Ventilatory mode change for transport	1.14 (0.52-2.51)	NS		
Change of PEEP for transport	0.76 (0.27-2.13)	NS		
Treatment modification for transport	2.66 (1.44-4.91)	0.002	2.81 (1.5-5.3)	0.001
Sedation for transport	3.05 (1.5-6.21)	0.003		NS
Neuromuscular blocker use for transport	0.93 (0.29-2.98)	NS		
Fluid challenge for transport	2.97 (0.92-9.54)	0.087		NS
**Quantitative data**	**Without AE**	**With AE**		**Univariate analysis ( *p *)**
**Patient data (Med (25th ,75th))**				
Age (yr)	57.5 [46–71.25]	59.5 [50.75-69.5]		NS
SAPS II	47 [34–61.5]	49 [34–64]		NS
**Transport data (average ± SD)**				
SOFA the day of transport	5 [2-8]	5 [3–8.75]		NS
MV duration before transport (days)	4.5 [2–12.25]	3.5 [2–9.75]		NS
No. of infusion pumps during transport	2 [1-3]	2 [2,3]		NS

*VAC* = volume assist–control; *BIPAP* = bi-level positive airway pressure; *PSV* = pressure support ventilation; *FiO*_*2*_ = fraction of inspired oxygen; *PEEP* = positive end-expiratory pressure; *SOFA* = Sepsis-related Organ Failure Assessment; *SAPS II* = Simplified Acute Physiology Score; *MV* = mechanical ventilation; *SD* = standard deviation; *OR* = odds ratio; *CI* = confidence interval; NS = not significant.

#### Major patient-related adverse events

Forty-four major patient-related AE occurred during IHT (16.8% of transports): 23 cases of oxygen desaturation (8.8%), 1 case of extubation (0.4%), 1 case of accidental central venous catheter removal (0.4%), and 13 cases of hemodynamic instability (5%) requiring increased vasopressor doses in 5 cases (1.9%). With univariate analysis, risk factors for major patient-related AE were: norepinephrine before transport (OR = 4 [1.8-8.8]; *p* = 0.001), dobutamine before transport (OR = 2.7 [1.1-6.7]; *p* = 0.041), treatment modification for transport (OR = 3 [1.4-6.4]; *p* = 0.006), and particularly, fluid infusion (OR = 3.8 [1.1-13.4]; *p* = 0.05) and hemodynamic reason for MV (OR = 3.2 [1.1-9.8]; *p* = 0.047). With multivariate analysis, parameters that predicted major patient-related AE during transport were norepinephrine before transport (OR = 4.4 [1.9-10]; *p* < 0.001) and treatment modification for transport (OR = 3.3 [1.5-7.4]; *p* = 0.001).

Considering their clinical importance, we also more specifically studied risk factors for oxygen desaturation: norepinephrine before transport (OR = 6.1 [2.3-16.1]; *p* < 0.0001), treatment modification for transport (OR = 6.1 [2.3-16]; *p* < 0.0001), and more than four infusion pumps (OR = 5 [1.1-23.8]; *p* = 0.04); and risk factors for hemodynamic instability: dobutamine before transport (OR = 8.5 [2.3-31]; *p* = 0.001) and fluid infusion for transport (OR = 11.9 [2.3-62]; *p* = 0.003).

### Equipment-related incidents

Equipment-related incidents are important to focus on because they are common (observed in 86 IHT (32.8%) in our study) and may be a target for prevention of AE. In 52 cases (19.8% of IHT), equipment-related incidents had no consequence on the patient. These equipment-related incidents were associated in univariate analysis with more than four infusion pumps, sedation before transport, PEEP > 6 cmH_2_O, treatment modification for transport, and fluid infusion for transport. They were less frequent if the junior physician was experienced (more than 6 months full-time training in ICU) or if he or she was an anaesthetics trainee. With multivariate analysis, risk factors for equipment-related incidents were: sedation before transport (OR = 2.3 [1.3-4.1]; *p* = 0.007) and PEEP > 6 cmH_2_O (OR = 2.1 [1.2-3.8]; *p* = 0.01).

Limiting equipment-related incidents is important, because they are associated with an increase in patient-related AE. With univariate analysis, these equipment-related incidents affecting the patient were associated with: number of infusion pumps, duration of CT scan, duration of transport, incidents with airway equipment, and incidents with infusion pumps. With multivariate analysis, only incidents with airway equipment (OR = 5.6 [2.7-11.7]; *p* < 0.0001) and duration of transport (OR = 1.04 [1.02-1.06]; *p* = 0.001) were associated with patient-related AE.

### Ventilator-associated pneumonia

In patients who suffered AE during transport, there was no increased incidence of ventilator-associated pneumonia compared with patients with IHT without AE (OR = 1.14 [0.7-1.9]; *p* = 0.7), whichever the type of AE (total events, patient-related AE, major patient-related AE, or equipment-related incidents during transport).

### ICU length of stay and time spent on MV

In our study, no difference in ICU length of stay (17.5 days [8–25.25] vs. 15 [8.75-28], *p* = 0.55) or the time spent on MV (13.5 days [6–23.25] vs. 10 [6-20], *p* = 0.38) was found between patients with or without patient-related AE during IHT.

## Discussion

The study objectives were to determine the frequency of AE during transport, the elements that predict the risk of IHT of critically ill patients, and the improvements to be put in place in our ICU.

### Incidence of adverse events

In our cohort of 262 IHT, we showed that AE occurred during 45.8% of IHT. Twenty-six percent of these IHT resulted in patient-related AE. Sixty-four percent of these patient-related AE were major (16.8% of transports) requiring medical intervention during transport.

Our cohort is one of the largest cohorts of IHT of patients on mechanical ventilation (262 IHT). Only two published series are larger: Lahner studied a cohort of 452 IHT of adults and children [9], and Kue recently published a retrospective study of 3,358 IHT [14]. This last study reported few AE (59 events, 1.7%) but only very serious patient AE were recorded. In our study, we identified a higher number of AE during IHT. This is however similar to the incidence reported in the literature: up to 68% of transports depending on the series [8-11,14,16,19-21]. The definition of AE is the most important confounding factor. In this study, we followed the most common definitions of AE [13]. Thus, our high incidence of AE despite respect of the protocol and practical training of junior physicians may be explained by the thorough recording of AE. Certainly some of these incidents (including “line, tube, and drain” incidents) can occur independently of IHT, but this AE incidence is lower than the incidence of AE during IHT. Many are preventable through proper preparation [22,23]. Thus, the team carrying the patient must be prepared for the occurrence of these incidents [24].

Equipment-related incidents represented the majority of incidents, but AE with an impact on the patient still occurred in 26% of IHT, which is similar to literature data (17-33% of transports) [9-11,19]. Our series includes only one accidental extubation and no cardiac arrest during IHT, unlike other studies [8-11,16,20]. This low percentage of very severe AE may be due to the presence of an experienced porter and perhaps also to more care and attention being paid to transport modalities under the study conditions.

No coordination problem with the radiology department was noted with specific daily time slots reserved for ICU patients. Synchronization with the radiology department is organized by our porters. Thus, our ICU patients wait a minimum of time in the radiology department during IHT.

### Risk factors of these AE

We have shown that AE occurred in 45.8% of IHT. Risk factors were fluid challenge for IHT, PEEP > 6 cmH_2_O, and sedation before transport. Twenty-six percent of these IHT resulted in patient-related AE. Risk factors specific for these patient-related AE were PEEP > 6 cmH_2_O before transport and treatment modification for transport. Sixty-four percent of these patient-related AE were major (16.8% of transports) with catecholamine administration and treatment modification for transport identified as predictive factors. In our study, AE during transport did not increase the risk of ventilator-associated pneumonia, ICU length of stay, or time spent on MV.

The male to female ratio was 2:3 (men = 129, women = 55) comparable to the proportions usually found in our ICU. Patient’s sex was not a risk factor for the occurrence of an AE during IHT. The repetition of IHT for the same patient was not a risk factor for AE during IHT. Each transport of the same patient is associated with a similar level of risk.

We conducted our study only for one type of transport (to CT scan) to exclude risk factors related to the radiology procedures and to identify risk factors related to the patient (and not to diseases or invasive techniques). Few IHT were performed after working hours. The time of day or day of the week had no influence on AE during IHT. The severity of patients and invasive devices are not predictive of AE during IHT. Even if this was assumed in other studies [11], it has not yet been demonstrated. We found sedation of the patient before transport, PEEP > 6 cmH_2_O, and the need for fluid infusion for transport to be risk factors for any AE during IHT. Sedation of the patient as high PEEP is a known risk factor in the literature [8-11,16,20] but not treatment modification identified in our study as a risk factor for patient-related AE. It is possible that transport was undertaken too early after treatment modification with insufficient time for stabilization, leading to a direct risk to the patient. Treatment modifications were primarily increase in sedation or fluid challenge, which should perhaps change our attitude. However, patient severity was not associated with AE during IHT. The number of infusion pumps was predictive of AE during IHT in univariate but not multivariate analysis, which also was shown by Doring [19]. This may be related to the fact that we limit the number of infusion pumps during transport in our protocol. We listed many equipment-related incidents during transport, which were usually battery problems or improperly set alarms (high pressure on ventilator). We used second- and third-generation respirators as recommended for these types of IHT [25]. Third-generation respirator was used preferentially if the patient was treated for ARDS or if ventilatory mode of the patient before IHT was PSV. The material used does not explain AE (no statistical difference). Experienced or anaesthetist junior physicians had fewer equipment-related incidents than the other junior physicians, because they are more familiar with the equipment used and may better anticipate potential problems with these devices. Most of these equipment-related incidents appear to be preventable, particularly those affecting the transport ventilators. They should be avoided by proper preparation and checks before IHT. Good coordination between the radiology team and the ICU is essential, because duration of transport also is associated with patient-related AE, a point already shown in other studies, such as those by Lahner or Smith [9,26].

AE during transport were not significantly associated with a higher risk of ventilator-associated pneumonia compared with patients transported without AE, perhaps because of a too small number of patients included in our study. This result differs from that of the study by Bercault, which compared patients with and without IHT [15]. Bercault’s study included all IHT, including IHT to MRI, coronary angiography, and arteriography (33/158 transports) with a much longer length of transport and a more systematic strict supine positioning. In this study, the risk of ventilator-associated pneumonia was associated with IHT [15]. However, this association is particularly difficult to interpret, because patients who require IHT have been shown to be more serious and to have a longer ICU and hospital length of stay, which are well-known risk factors of ventilator-associated pneumonia [27,28]. This reason also explains why we chose an observational design for our study. Using a randomized, control design or matching between patients with or without IHT to study these parameters would have explored more the consequence of the procedure than the effect of IHT. Of course, AE as recorded in our study can occur when a patient remains in his ICU room. Comparison in AE frequency during these two periods requires further study.

Finally, AE during IHT had little impact on patient outcomes; we did not find that an AE during the IHT entailed consequences for time spent on MV or ICU length of stay.

### Consequences for our practices

Our study had a direct impact on our practices. It allowed us to identify most common AE, to overcome the most frequent errors, to check the equipment (replacement of batteries for example), and to implement a reproducible protocol for IHT, with training of junior physicians, but also of nurses [29-31]. Particular attention should be paid to the risk factors that were found in our study. Our single-center study can probably be extrapolated to other centers, because our protocol was performed in accordance with the widely used guidelines of Warren et al. [4]. Our conclusions may be useful to other ICUs because they are consistent with recent guidelines [13], and the points highlighted are probably encountered in other services. The main consequence we have drawn from our study is to increase the size of the transport team, including an ICU nurse to help the junior physician to deal with the complexity of ICU patient. Using such a three-person transport team has recently been recommended by Fanara [13]. However, the beneficial effect of such a three-person transport team in reducing AE during IHT should be evaluated by a further study.

### Study limitations

Several limitations of this study should be noted. The main bias of our study is the fact that some patients in need of a procedure requiring transport may have had their transport denied by senior physicians because it was estimated to be too dangerous given the severity of the patient. Some data have not been included in our study, such as the reasons for CT scan. These data would have allowed more detailed analyses regarding “urgent” or “routine” CT scan and the relevance of the CT scan and therefore transport. Our study may not be extrapolated to all ICUs, because some diseases (such as neurosurgical disease) or certain types of invasive techniques (such as the measurement of intracranial pressure) are poorly represented in our study.

Our study has focused on patients and their immediate environment. Because the healthcare delivery is very complex, both provider and systems (two major components beyond patients in healthcare delivery) need to be modified to reach good outcome for both patients and organizations [32]. A more systematic approach would perhaps show other risk factors that are accessible to improvement.

## Conclusions

This study confirms that the IHT of ICU patient leads to a significant number of AE. Although, in our study, AE have not had major consequences on the rest of patient stay, efforts should be made to decrease their incidence. These complications should be prevented by using standardized procedures and medical surveillance throughout the transport. Physicians have to evaluate the risk-benefit ratio of each transport, with particular attention to the indication for IHT.

## Abbreviations

ICU: Intensive care unit; IHT: Intrahospital transport; AE: Adverse event; MV: Mechanical ventilation; PEEP: Positive end-expiratory pressure; SAPS: Simplified acute physiology score; SOFA: Sequential organ failure assessment; FiO2: Fraction of inspired oxygen; SpO2: Pulse oximetry; SD: Standard deviation; OR: Odds ratios; COPD: Chronic obstructive pulmonary disease; ALI: Acute lung injury; ARDS: Acute respiratory distress syndrome; VAC: Volume assist–control; BIPAP: Bi-level positive airway pressure; PSV: Pressure support ventilation.

## Competing interests

We declare no conflict of interest or financial interests relevant to this manuscript.

## Authors’ contribution

EP, SN, RF, and DM designed the study. EP and JP collected data. EP, MJG, and DM wrote the manuscript, and all authors participated in its critical revision. EP had full access to all data in the study and had final responsibility for the decision to submit for publication. All authors read and approved the final manuscript.

## Supplementary Material

Additional file 1 Protocol of intra-hospital transport. Click here for file
