# Supplementary material for: Adverse events during intrahospital transport of critically ill patients: incidence and risk factors
Source: Ann Intensive Care. 2013 Apr 12;3:10. doi: 10.1186/2110-5820-3-10 (PMC3639083; doi:10.1186/2110-5820-3-10)
Supplement: Additional file 1 — Protocol of intra-hospital transport. [file 2110-5820-3-10-S1.doc]

Additional file 1 : Protocol of intra-hospital transport

### Required Equipment

 Sufficient medication,

 Airway equipment : bag + valve + mask, adapted portable mechanical ventilator with O2 and electrical reserves.

 Mechanical ventilation adapted to the patient (alarm and monitoring of tidal volume and insufflation pressure, trigger),

 Monitor (with ECG, IAP, SpO2) with electrical reserves and with Alarms adjusted and activated.

 Emergency case

 A phone and the number of the responsible senior physician.

### Preparing the patient

 Stop nutrition during transport

 All bags emptied before departure

 Lines, cables and drainage tubes (Heimlich chest tube valve, abdomen, bladder) unclamped, functional, secure, untangled and transportable

 Limit the number of infusion pumps as much as possible

 Aspirate the patient before departure and check the cuff pressure of endotracheal tube

 Head raised if possible (to prevent intracranial hypertension and ventilator-associated pneumonia)

 Prepare medication (emergency, sedation, analgesia, paralysing agents), fluid loading solutions

 Route for venous access isolated and secured (quick injection, administration of vasopressors)

### Transport organisation

 Confirmation of the timetable for the procedure

 Transport route clear, lifts and emergency room available

 Operational equipment (O2 and electrical supplies, ventilator, aspirator) for continuous treatment at sites of procedure

### Preparing the mechanical ventilator

 Check the transport ventilator with a mechanical lung (pressure alarms, parameters, exhalation valve)

 Respect mechanical ventilation parameters and the PEEP previously prescribed to the patient

 Connect the transport respirator a few minutes before leaving

 Adjust the alarms

### During transport

 Continuous cardiac and blood pressure monitoring and SpO2 monitoring

 A minimum of two escorts available (a porter and the junior physician responsible for the patient who is familiar with the patient’s medical history)

 Stop if any alarm sounds

### During CT-scan

 Connect the oxygen to the wall

 Connect monitor and infusion pumps to the mains

 Connect the chest tube on the vacuum if necessary
